# Messenger RNA expressing PfCSP induces functional, protective immune responses against malaria in mice

**DOI:** 10.1038/s41541-021-00345-0

**Published:** 2021-06-18

**Authors:** Katherine L. Mallory, Justin A. Taylor, Xiaoyan Zou, Ishita N. Waghela, Cosette G. Schneider, Michael Q. Sibilo, Neeraja M. Punde, Leah C. Perazzo, Tatyana Savransky, Martha Sedegah, Sheetij Dutta, Chris J. Janse, Norbert Pardi, Paulo J. C. Lin, Ying K. Tam, Drew Weissman, Evelina Angov

**Affiliations:** 1 Walter Reed Army Institute of Research, Silver Spring, MD USA; 2 Parsons Corporation, Centreville, VA USA; 3 The Geneva Foundation, Tacoma, WA USA; 4 Naval Medical Research Center, Silver Spring, MD USA; 5 Henry M. Jackson Foundation for the Advancement of Military Medicine, Bethesda, MD USA; 6 Oak Ridge Institute for Science and Education, Oak Ridge, TN USA; 7 General Dynamics Information Technology, Falls Church, VA USA; 8 Leiden University Medical Center, Leiden, the Netherlands; 9 University of Pennsylvania, Philadelphia, PA USA; 10 Acuitas Therapeutics, Vancouver, BC Canada

**Keywords:** Vaccines, RNA vaccines, Infectious diseases, Malaria, Microbiology

## Abstract

Human malaria affects the vast majority of the world’s population with the *Plasmodium falciparum* species causing the highest rates of morbidity and mortality. With no licensed vaccine and leading candidates achieving suboptimal protection in the field, the need for an effective immunoprophylactic option continues to motivate the malaria research community to explore alternative technologies. Recent advances in the mRNA discipline have elevated the long-neglected platform to the forefront of infectious disease research. As the immunodominant coat protein of the invasive stage of the malaria parasite, circumsporozoite protein (PfCSP) was selected as the antigen of choice to assess the immunogenic and protective potential of an mRNA malaria vaccine. In mammalian cell transfection experiments, *PfCSP* mRNA was well expressed and cell associated. In the transition to an in vivo murine model, lipid nanoparticle (LNP) encapsulation was applied to protect and deliver the mRNA to the cell translation machinery and supply adjuvant activity. The immunogenic effect of an array of factors was explored, such as formulation, dose, number, and interval of immunizations. *PfCSP* mRNA-LNP achieved sterile protection against infection with two *P. berghei* PfCSP transgenic parasite strains, with mRNA dose and vaccination interval having a greater effect on outcome. This investigation serves as the assessment of pre-erythrocytic malaria, *PfCSP* mRNA vaccine candidate resulting in sterile protection, with numerous factors affecting protective efficacy, making it a compelling candidate for further investigation.

## Introduction

Malaria is an infectious disease older than human history^1^. In 2018, there were 228,000,000 clinical cases of malaria and 405,000 deaths worldwide, demonstrating its significance as a global health threat^2^. Strategies for infection control of this global pathogen include a variety of approaches, such as vector control, antimalarial drugs, and vaccines^2^. As the vector develops insecticide resistance and the parasite exhibits signs of drug resistance, efforts are ongoing to develop a potent, effective malaria vaccine^3^. To date, the most clinically advanced malaria vaccine candidate, RTS, S/AS01, has achieved protection against clinical endpoints in the field; however, the observed protective efficacy reaches 30–40% before diminishing over time, reinforcing the need for a more potent and sustainable immunoprophylactic option^4^. RTS, S is a hepatitis B surface antigen, virus-like particle fusion protein displaying the carboxy-terminal portion and 18 NANP copies of the highly repetitive region of the *Plasmodium falciparum* circumsporozoite protein (PfCSP), the dominant coat antigen of the invasive sporozoite-stage parasite^2^. Notably, the *falciparum* species of *Plasmodium* protozoa is the most prevalent and most lethal.

Second-generation PfCSP vaccine candidates have transitioned to evaluate other nanoparticle display platforms, such as self-assembling protein nanoparticles^5,6^ and the tobacco mosaic virus platform^7^, for high-density epitope display. To broaden immunity, larger segments of PfCSP including the N-terminal sequence, not present in RTS, S, have been expressed either in soluble form^8–12^, as genetic fusions on virus-like particles, SpyCatcher^13^, or as chemically conjugated on virus-like Qβ particles^14^. Lastly, delivery of PfCSP plasmid DNA by electroporation has been shown to effectively drive a potent cellular immune response^15,16^. To produce a more agile and efficacious vaccine, novel technologies capable of harnessing both humoral and cellular responses, such as messenger ribonucleic acid (mRNA) need to be evaluated.

Recent advances in mRNA technology for stable, targeted antigen expression make this platform an appealing alternative to conventional vaccine approaches^17^. mRNA enables the encoded antigen to be expressed within the cells without altering the host cell genome or requiring access to the nucleus^18,19^. While some success has been achieved with naked delivery of mRNA, the majority of recent products use nucleoside-modified mRNA to ablate innate immune activation and are co-administered with a molecular carrier^20^. These carriers serve an array of functions, including protection from degradation, immunostimulation, and efficient intracellular delivery^20,21^. Lipid nanoparticles (LNP) are one of the carrier methods with positive safety outcomes in the clinic and potency when applied to mRNA^22,23^. A single dose of mRNA-LNP formulation is capable of inducing high levels of immune responses, however, additional immunizations are not uncommon^24–27^. In pursuing an immunogenic, protective vaccine against malaria, we designed two *PfCSP* mRNA constructs for evaluation. In vitro methods were used to assess protein expression in mammalian cells transfected with *PfCSP* mRNA. To address protection and delivery of the mRNA, *PfCSP* mRNA was encapsulated in LNP for investigation in vivo. We found the translated PfCSP proteins to be well expressed in mammalian cells and *PfCSP* mRNA-LNP to be highly immunogenic, yielding protective responses against two murine transgenic parasite infectivity models.

## Results

### *PfCSP* is expressed in mammalian cells and remains cell associated

Expression of the *PfCSP* mRNA (TriLink) was assessed in transfected mammalian cells. Translated PfCSP protein was successfully detected by fluorescence microscopy (Supplementary Fig. 1). Fluorescent images depict the nuclear stain (DAPI) alone, detection of PfCSP alone, and the overlay (Supplementary Fig. 1). Negative control images *sans PfCSP* mRNA exhibited no detection of PfCSP under identical transfection and detection conditions (Supplementary Fig. 1). Calculation of the FITC detection area relative to the DAPI detection area quantified the level of PfCSP within a field normalized to the size and number of cells (Supplementary Fig. 1). The FITC-conjugated PfCSP detection was significant under *PfCSP* mRNA transfection conditions, and relative to the negative control, which included cells exposed to the transfection reagents in the absence of mRNA. To determine if the protein was secreted or accumulated in the cell, *PfCSP* mRNA transfected cell culture supernatant and pellet samples were collected for western blot analysis (Supplementary Fig. 1). Samples were harvested at 8, 24, and 48 h time points following transfection. Recombinant PfCSP (r-PfCSP) was included on each blot as a blotting control and as a reference for band intensity. The r-PfCSP migrated lower as it has reduced numbers of the central repeat motif, NANP, compared to native protein. Mock transfection samples mimicked the experimental conditions, but lacked mRNA, resulting in no detection of PfCSP (Supplementary Fig. 1). Similarly, PfCSP was not detected in the culture supernatant of cells transfected with *PfCSP* mRNA, indicating that PfCSP was not secreted even in the context of a native signal sequence. A major band was detected migrating at ~60 kDa in the pellet fraction of CHO cells transfected with *PfCSP* mRNA (Supplementary Fig. 1). Full-length native PfCSP has an expected molecular weight of ~41.1 kDa and migrates anomalously on SDS-PAGE due to the high copy repeats in the central region. The relative density of the major band was calculated using the 10 ng r-PfCSP (expected molecular weight is ~33 kDa, actual gel migration is ~50 kDa) as reference. From this analysis, protein levels appeared to peak between 8 and 24 h, with a dramatic reduction observed in total protein by 48 h (Supplementary Fig. 1).

To examine the in vitro transfection of mRNA-LNP, CHO cells were transfected with *PfCSP* mRNA–LNP1 (TriLink) and *PfCSP* mRNA-LNP (UPenn) using TransIT reagent alone (no Boost reagent). Samples were harvested at 48-h time point following transfection. 10 ng and 3 ng r-PfCSP were used as reference. A positive control with Boost reagent and TransIT reagents exhibited detection of PfCSP in the pellet fraction (Supplementary Fig. 1). At 48-h, PfCSP was detected in the pellet fraction of CHO cells transfected with either *PfCSP* mRNA-LNP1 (TriLink) or *PfCSP* mRNA-LNP1 (UPenn) (Supplementary Fig. 1). A qualitative difference was observed between the expression levels of PfCSP transfected with UPenn *PfCSP* mRNA-LNP1, which was relatively higher than cells transfected with *PfCSP* mRNA-LNP1 (TriLink).

### mRNA-LNP proves a highly immunogenic platform for *PfCSP* delivery

**Fig. 1:**
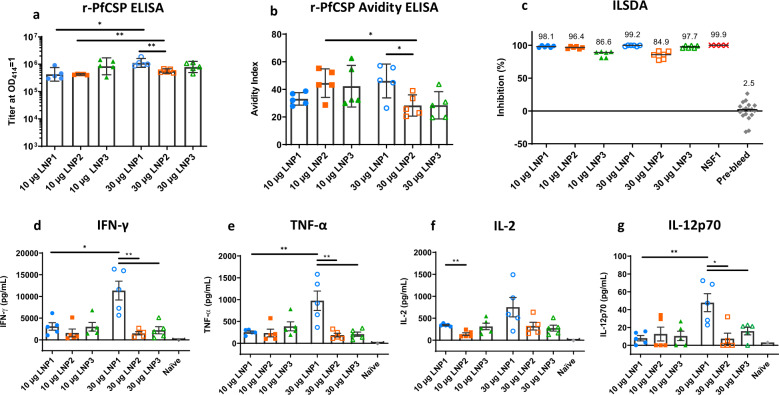
*PfCSP* mRNA-LNP induce potent functional antibodies and cytokine responses. *PfCSP* mRNA (TriLink) was encapsulated in three different LNP formulations. BALB/c were injected with 10 µg and 30 µg dose of *PfCSP* mRNA-LNPs in a 3 week interval prime:boost regimen (*N* = 5 per group). Serum and splenocytes were collected two weeks following the boost injection. **a** PfCSP antibody responses were determined by r-PfCSP titration ELISA and reported as the geometric mean (GM) with 95% confidence intervals (CI). **b** Avidity indices were calculated using a chaotrope-based avidity r-PfCSP titration ELISA and reported as the GM with 95% CI. **c** Functional activity of the antibodies was quantified by ILSDA using pooled mouse serum of each group. Inhibition was reported as the mean of assay replicates and calculated relative to the mean of sporozoite invasion in the absence of mouse serum. For **d** IFN-γ, **e** TNF-α, **f** IL-2, and **g** IL-12p70, splenocytes were incubated in the presence of a PfCSP overlapping 15-mer peptide pool. Cell culture supernatants were harvested after 48 h and cytokine concentrations quantified by MSD. Data are reported as the mean and standard error of the mean (SEM). All statistical analyses were performed using a Mann Whitney test (*p < 0.05; **p < 0.01).

As a well-characterized carrier system with positive clinical outcomes^17,24,25,28^, LNPs were selected for mRNA encapsulation. Several formulations were tested with *PfCSP* mRNA (TriLink) to confirm that an antigen-specific response could be elicited. *PfCSP* mRNA was encapsulated in LNP incorporating three different lipids proprietary to Acuitas Therapeutics. The formulations were delivered in a two-immunization regimen at a three-week interval. Each LNP condition included a low and high-dose mRNA group (10 µg and 30 µg, respectively). Final sample collections were performed two weeks following the final immunization. Humoral responses were assessed using an r-PfCSP IgG titer ELISA (Fig. 1a). Generally, the titers were high with a statistically significant difference between the low and high dose groups of LNP1 and LNP2. In addition, the high dose of LNP1 induced superior titers compared to the LNP2 high dose group. Responses from naïve, pre-immune serum did not achieve the lower limits of detection in the r-PfCSP IgG titer ELISA assay (data not shown).

To assess the overall strength of antibody–antigen interactions, antibody avidity measurements were made using an r-PfCSP avidity ELISA (Fig. 1b). Similar to the observation made for r-PfCSP IgG titer, the avidity indices for the high dose LNP1 group were higher relative to the LNP2 high dose group. Interestingly, the high dose groups in LNP2 and LNP3 yielded responses with lower avidity indices compared to their respective low dose counterparts. Nominally, since the quality and quantity of the antibody response influence functional outcomes, to this end, the inhibition of liver stage development assay (ILSDA) was performed to detect antibody functionality directly against the *P. falciparum* malaria parasite in vitro^39^. For this experiment, individual mouse sera within each group were pooled and tested at a 1:40 dilution (Fig. 1c). Generally, sporozoite inhibition was high for all groups. However, the two groups with the highest average of inhibition were the LNP1 low and high dose groups at 98.1% and 99.2% inhibition, respectively. This finding is of importance as it demonstrates the potential for *PfCSP* mRNA-LNP to induce antibodies that inhibit falciparum malaria sporozoites invasion and development in human hepatocytes.

To assess cellular immune responses, splenocytes were isolated 2 weeks following the final immunization and stimulated with a PfCSP overlapping 15-mer peptide pool. IFN-γ ELISpot assays were undertaken, however, the high sensitivity of the assay yielded a number of antigen-specific spot-forming cells that could not be counted under the assay conditions (data not shown). As an alternative approach to quantify bulk cytokines, the Mesoscale Discovery (MSD) multiplex assay was performed on supernatants from splenocyte cell cultures stimulated with the PfCSP overlapping 15-mer peptide pool. Cytokine concentrations were quantified for IFN-γ, IL-1β, IL-2, IL-4, IL-5, IL-6, KC/GRO, IL-10, IL-12p70, and TNF-α. In addition to the individual mouse splenocyte samples from each group, a splenocyte pool of five naïve mice served as a negative control for auto-reactivity induced by the PfCSP peptide pool. Th2 skewed cytokine levels (IL-4 and IL-5) in the panel were detectable, but low (Supplementary Fig. 2). The pro-inflammatory skewed cytokines (IL-10, IL-1β, IL-6, and KC/GRO) indicated modest significance between groups in favor of high dose *PfCSP* mRNA-LNP1 formulation (Supplementary Fig. 2). Nominally, the presence of IL-10 is indicative of a highly potent immune response as regulatory pathways operate to control it^29,30^. The Th1 skewed cytokines within the panel (IFN-γ, TNF-α, IL-2, and IL-12p70) were high with IFN-γ being specifically high (Fig. 1d, e, f, g). The LNP1 high dose group achieved significantly higher IFN-γ, TNF-α, and IL-12p70 concentrations relative to the LNP2 and LNP3 high dose groups. LNP1 low dose group had significantly higher IL-2 responses relative to the LNP2 low dose group, a hallmark cytokine secreted by immune cells and central in a potent, specific cell-mediated response.

### Boost immunizations of *PfCSP* mRNA-LNP1 are necessary to induce potent immune responses

Dose number and immunization schedule can have an effect on immunity against malaria^31^. As the mRNA vaccine field has expanded, a range of dosing strategies have been evaluated, including single-dose regimens^24,26,32^. To explore a single-dose regimen and an extended interval between doses for *PfCSP* mRNA (TriLink), mice were immunized either once with a 30 µg dose *PfCSP* mRNA-LNP1 (0–30 µg) or twice with 10 µg doses in a 3 week interval (0,3 - 10 µg) or 4 week interval (0,4 - 10 µg). Blood and splenocyte samples were collected following the final (or only) immunization (Supplementary Fig. 3). All groups achieved detectable levels of PfCSP-specific antibodies in an r-PfCSP IgG ELISA (Supplementary Fig. 3). However, the single 30 µg dose group titers were significantly lower compared to the twice dosed with 10 µg at 3- and 4-week intervals. No significant differences in antibody responses were detected between the three- and four-week interval groups. When assessed for antibody functionality by ILSDA, boosted samples induced high levels of inhibition, while the single-dose group did not achieve inhibition above the background level of naïve mouse serum (Supplementary Fig. 3). Notably, some differences in total responses were observed between the 10 µg dose groups of *PfCSP* mRNA-LNP1, reported in Fig. 1 and Supplementary Fig. 2, with the r-PfCSP IgG titers and IFN-γ responses approximately threefold higher in the latter, compared to the former. While the experimental conditions between these two groups were essentially identical, some differences in the lots of mRNA, LNP, and/or mice may account for this experiment-to-experiment variation.

To further characterize the difference in immune responses elicited by *PfCSP* mRNA-LNP1, cellular responses were examined using an IFN-γ ELISpot (Supplementary Fig. 3) and the MSD multiplex assay (Supplementary Fig. 3). Although the ELISpot assay conditions were modified to capture high numbers of spot-forming cells, one sample from the four-week interval group was outside the upper limit of detection. Since the data set was incomplete, statistical analysis was not performed on the ELISpot data. Despite the broad range of concentrations, MSD analysis was able to capture all responses. Three of the prominent Th1-skew cytokines, IFN-γ, TNF-α, and IL-2 are shown in Supplementary Fig. 3, with the remaining reported in Supplementary Fig. 4. The 4-week interval group had significantly higher IFN-γ, TNF-α, and IL-2 concentrations detected compared to the single-dose group (Supplementary Fig. 3). Due to the variability in responses in the three-week interval group, this group only achieved significance compared to the single-dose group with TNF-α, when exclusively observing the Th1 cytokines within the panel. As in the previous study, the Th2 skewed cytokines (IL-4 and IL-5) concentrations were low (Supplementary Fig. 4). Most of the pro-inflammatory cytokines (IL-10, IL-1β, and IL-6) were present and some with significant differences detected between the 0–30 µg (single dose) and 0, 4 week- 10 µg groups (Supplementary Fig. 4). The KC/GRO and IL-12p70 cytokines were non-specifically detected, since equivalent levels were measured in the naïve mouse group (Supplementary Fig. 4). Across all cytokines, the twice-dosed groups had no significant difference in detection compared to each other.

### *PfCSP* mRNA-LNP1 induces protection in mice

To elucidate the protective potential of *PfCSP* mRNA-LNP1, a rodent malaria challenge study was undertaken. Since human malaria parasites are non-infectious in mice, transgenic rodent parasites expressing the human malaria antigen of interest or humanized mice are required to assess protective responses in rodent models. The animal model selected for this study was a rodent malaria (*P. berghei* ANKA) transgenic parasite line (Pb-PfCSP) expressing the *P. falciparum* NF54/3D7 allele of *csp* at the *P. berghei* (*pb*) *csp* locus and under the *pbcsp* promoter. Since there is limited data on sporozoite infectivity of this transgenic line in BALB/c mice, a series of intravenous sporozoite dose titration experiments were performed (Supplementary Fig. 5). The dose range (300, 1000, and 3000 sporozoites) was selected based on data reported from a wild-type *P. berghei* ANKA strain^33^. All mice across three independent experiments became infected at all doses, demonstrating overlapping, reproducible and high levels of infectivity.

**Fig. 2:**
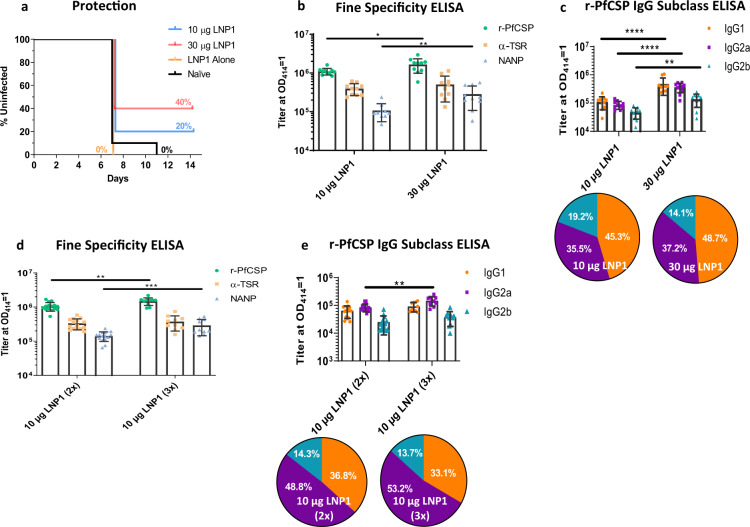
*PfCSP* mRNA-LNP1 elicits a dose-dependent response in BALB/c. A 10 µg and 30 µg dose *PfCSP* mRNA-LNP1 (TriLink) prime:boost regimen at a 3-week interval was tested in a Pb(ANKA)-PfCSP (NF54/3D7) transgenic challenge experiment (*N* = 10 per group) with 1000 sporozoites injected intravenously. **a** Kaplan–Meier survival curves report the percent survival of infected mice. Efficacy was calculated against the negative controls (LNP1 alone and naïve controls). Survival was defined as mice without detectable parasitemia by thin blood smear by day 14. **b** Fine specificity ELISAs were performed to quantify IgG responses to the r-PfCSP protein, the (NANP)_6_ repeat region, and the α-TSR domain peptides. Data are reported as the GM with 95% CI. **c** To capture the IgG subclasses elicited by *PfCSP* mRNA-LNP1, r-PfCSP ELISAs were performed and IgG1, IgG2a, and IgG2b OD 1.0 titers reported. Data in the bar graph are reported as the GM with 95% CI. Pie charts are representative of the relative response of each subclass. To assess the influence of an additional booster dose in a 3-week immunization regimen, mice were immunized in a prime:boost (2x) (*N* = 15 per group) and prime:boost:boost (3x) regimen (*N* = 10 per group). IgG was quantified for **d** r-PfCSP, α-TSR domain, (NANP)_6_ repeat peptide and **e** IgG subclass. All statistical analyses were performed using a Mann Whitney test (*p < 0.05; **p < 0.01; ***p < 0.001; ****p < 0.0001).

Having established the relative infectivity in the animal model, a challenge experiment was initiated. In this *PfCSP* mRNA-LNP1 protection study, a 30 µg dose, 10 µg dose, and LNP1 alone group were immunized twice with a 3-week interval (*N* = 10). Two weeks following the final immunization, blood samples were collected for antibody analyses. The challenge was performed by intravenous inoculation of 1000 Pb-PfCSP NF54/3D7 sporozoites. Mice were tested for blood-stage parasitemia by Giemsa stained thin blood smears on days 7, 8, 10, 11, and 14-post challenge. The kinetics of parasitemia detection are shown using Kaplan–Meier survival curves (Fig. 2a). Mice were sterile protected if no parasites were detected by day 14, the final experiment day. Vaccine efficacy of *PfCSP* mRNA-LNP1 was 40% for the high dose group and 20% for the low dose group, relative to unvaccinated challenge controls, which all developed a blood-stage infection. While no statistical differences were detected between the two dosage groups, the high dose group did achieve significance when compared against the challenge controls (*p* = 0.0387, Log-rank, Mantel–Cox). Because the two arms of the immune response, adaptive and innate, are highly intertwined, to adequately address the possible impact of the innate immune response on challenge outcomes, in subsequent studies, longer intervals between the final vaccination dose and the challenge will be evaluated, i.e., 4–6 weeks.

Antibody correlates of PfCSP vaccine-induced responses have included detection of antibodies against recombinant CSPs, the central NANP repeat and the C-terminal α-TSR regions^34,35^. Titer ELISAs were performed to detect the spectrum of IgG fine specificities to r-PfCSP, the C-terminal α-TSR domain, and the central repeat region (NANP). All coating antigens yielded high levels of antibody detection (Fig. 2b). r-PfCSP and NANP responses exhibited a significant dose-dependent increase between the vaccinated groups. To determine if a balanced or skewed IgG subclass response was induced by *PfCSP* mRNA-LNP1, subclass ELISAs were performed (Fig. 2c). In the high and low dose groups, IgG1 and IgG2a achieved higher titers than IgG2b. However, there was no significant difference between the IgG1 and IgG2a levels within each group or the IgG2a/IgG1 ratio between groups, indicating that the two dose regimen of *PfCSP* mRNA-LNP1 induces a balanced Th1/Th2 response. Unrelated to the similarity between subclass ratios, there was a significant increase in all subclass responses in the higher dose group relative to the lower dose group.

### A third dose of *PfCSP* mRNA-LNP1 boosts humoral responses

Additional vaccine doses enhance immunogenicity and similar effects have been reported for mRNA^36–38^. To evaluate if a third dose could boost immune responses, mice were injected either two or three times with 10 µg *PfCSP* mRNA-LNP1 at a three-week interval. Blood samples were collected the day prior to each immunization and two weeks following the final dose. Antigen-specific ELISAs were used to determine if an additional vaccine dose could increase ELISA titers to r-PfCSP, α-TSR domain, and the repeat region (NANP). Similar to the analysis performed between the two high dose (30 µg) and two low dose (10 µg) groups in Fig. 2b, antibody responses to the α-TSR domain were not boosted with a third dose of 10 µg *PfCSP* mRNA-LNP1 (Fig. 2d). However, the r-PfCSP and NANP responses were significantly boosted with the additional dose.

Next, we examined the effect of three versus two immunizations on antigen-specific IgG subclass titers (Fig. 2e). Overall, the IgG1 and IgG2a responses were enhanced in the three-immunization group, compared to the two-immunization group. Differences in IgG2b did not achieve statistical significance between the two groups. When comparing the IgG subclasses within the groups, the IgG1 and IgG2a levels, once again, were higher than IgG2b in both groups. In the two-dose group, the IgG1 and IgG2a levels were not significantly different from each other. However, in the three dose group, a *p* value of 0.0128 was achieved when comparing the IgG1 and IgG2a subclass responses, with IgG2a being higher. From these data, a three-dose immunization regimen appears to drive the immune response toward a Th1 bias. That said, we did not observe a significant difference in the IgG2a/IgG1 ratio between the two groups.

### Increasing the interval to 6-weeks yields improved protection

**Fig. 3:**
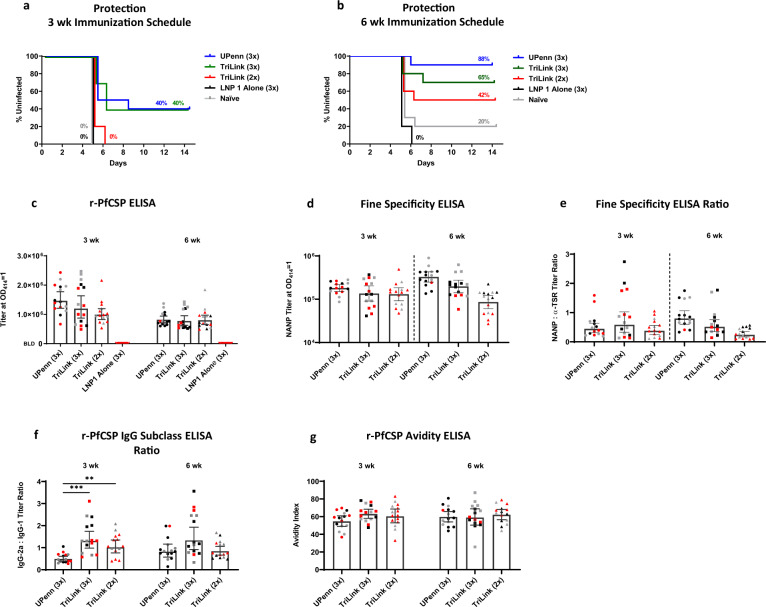
*PfCSP* mRNA-LNP1 induced responses in BALB/c. A 10 μg dose of *PfCSP* mRNA, TriLink and UPenn, prime:boost (2x) or prime:boost:boost (3x) regimen at a 3 week or 6 week interval was tested in a Pb(ANKA)-PfCSP (NF54/3D7) transgenic challenge model (*N* = 10 for all groups except LNP1 Alone, *N* = 5) with 1000 sporozoites injected intravenously, two weeks post final immunization. **a**–**b** Kaplan–Meier curves depict the percent survival of challenged mice with **a** 3 week and **b** 6 week immunization schedules and are extrapolated from the negative controls’ (LNP1 Alone and Naïve) survival percentage. Survival was defined as mice without detectable parasitemia by thin blood smear. PfCSP antibody responses were determined by **c** r-PfCSP ELISA, **d** fine specificity ELISA against the (NANP)6 repeat peptide, **e** the ratio of the (NANP)6 titer to the α-TSR titer, **f** the ratio of r-PfCSP IgG subclass titers for IgG2a and IgG1, and **g** r-PfCSP avidity ELISA (*N* = 15 UPenn 3x, TriLink 3x, TriLink 2x) (*N* = 8 LNP1 Alone). Uninfected, infected, and unchallenged mice are represented by black, red, and gray symbols, respectively. Data in the bar graphs are reported as the GM with 95% CI. All statistical analyses were performed using one-way ANOVA on ranks, Kruskal-Wallis post hoc Dunn’s multiple comparison test (** = *p* < 0.01; *** = *p* < 0.001).

To further assess immune responses induced by *PfCSP* mRNA-LNP, we evaluated two different mRNAs either obtained commercially (TriLink) or through collaboration (University of Pennsylvania) in parallel studies. These mRNAs were essentially identical in the *P. falciparum* CSP coding sequence, however differed in that the UPenn *PfCSP* mRNA was nucleoside-modified while the TriLink mRNA was not. Other sequence differences included proprietary UTRs and noncoding sequence regions. BALB/c mice vaccinated either three times with 10 µg mRNA at a 3-week interval or three times at a 6-week interval were challenged with 1000 *P. berghei* PfCSP NF54/3D7 transgenic parasites, as described above. For the 3-week interval, both the UPenn and TriLink *PfCSP* mRNAs yielded 40% of mice sterile protected. While in the 6-week interval cohorts, the UPenn *PfCSP* mRNA-LNP yielded high levels of sterile protection, with 88% efficacy against challenge controls, e.g., LNP1 alone and naïve (Fig. 3a, b). Antibody responses against each subunit, suggested no differences between the UPenn and TriLink *PfCSP* mRNAs administered at the 3-week or 6-week intervals (Fig. 3c). However, a qualitative difference in the ratio of NANP repeat responses relative to the C-terminal α-TSR suggested that the UPenn mRNA delivered at a 6-week interval yielded a higher ratio of NANP antibodies (Fig. 3d, e). No difference in IgG subclass or r-PfCSP antibody avidity was observed between groups at the 6-week interval (Fig. 3f, g). Differences observed in cellular cytokine levels may reflect differences in the nature of the mRNAs, i.e., UPenn *PfCSP* mRNA being nucleoside-modified and cellulose purified, while mRNA obtained from TriLink was nucleoside-unmodified and silica membrane purified (Supplementary Fig. 6).

### *PfCSP* mRNA-LNP induce protective responses in C57BL6 mice

**Fig. 4:**
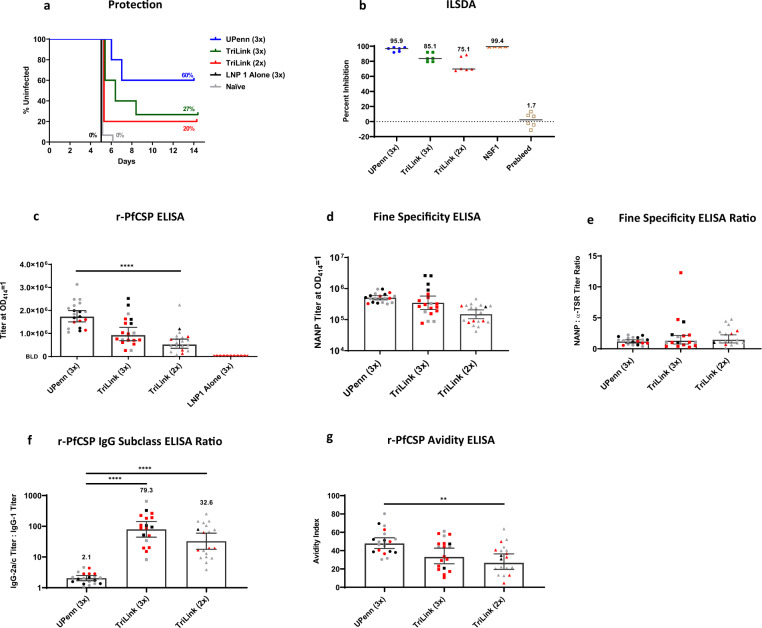
*PfCSP* mRNA-LNP1 induced responses in C57BL6. A 10 μg dose of *PfCSP* mRNA-LNP1, TriLink, and UPenn, prime:boost (2x) or prime:boost:boost (3x) regimen on a 3-week interval was tested against Pb(ANKA)-PfCSP (Wellcome) transgenic parasites (*N* = 15 per group for TriLink 3x and naïve controls) (*N* = 10, UPenn 3x) (*N* = 5 per group for TriLink 2x and LNP1 alone) with 1000 sporozoites injected intravenously, 2 weeks post final immunization. **a** Kaplan–Meier curves report the percent survival of challenged mice. Efficacy was calculated against the negative controls (LNP1 alone and naïve controls). Survival was defined as mice without detectable parasitemia by thin blood smear by day 14. **b** Functional activity of the antibodies was quantified by the ILSDA using pooled mouse serum of each group. Inhibition was calculated relative to the mean of sporozoite invasion in the absence of mouse serum. Data are reported as the mean of assay replicates. **c** r-PfCSP ELISA (*N* = 20 per group for UPenn 3x, TriLink 2x) (*N* = 19, TriLink 3x) (*N* = 10, LNP1 alone), **d** fine specificity ELISA against the (NANP)_6_ repeat peptide (*N* = 20 per group for UPenn 3x, TriLink 2x) (*N* = 19, TriLink 3x), **e** fine specificity ELISA are reported as the ratio of the (NANP)_6_ titer to the α-TSR titer (*N* = 20, UPenn) (*N* = 17 per group for TriLink 3x, TriLink 2x), **f** the ratio of r-PfCSP IgG subclass titers for IgG1 and IgG2a (*N* = 20, UPenn 3x) (*N* = 18, TriLink 3x) (*N* = 19, TriLink 2x) and **g** r-PfCSP avidity ELISA (*N* = 20, UPenn 3x) (*N* = 19 per group for TriLink 3x, TriLink 2x). Uninfected, infected, and unchallenged mice are represented by black, red, and gray symbols, respectively. Data in the bar graph are reported as the GM with 95% CI. All statistical analyses were performed using one-way ANOVA on ranks, Kruskal–Wallis post hoc Dunn’s multiple comparison test (**p < 0.01; ****p < 0.0001).

We next examined *PfCSP* mRNA-LNPs in C57BL6 mice to assess the generalizability of the induced immune responses. Mice were vaccinated 3 times with 10 µg mRNA at a 3-week interval and challenged by the intravenous route with 1000 infectious *P. berghei* PfCSP Wellcome strain transgenic parasites, two weeks after the final dose. While the C57BL6 mice were challenged with a heterologous strain, and BALB/c mice were challenged with homologous parasite transgenic strain relative to PfCSP, the homology between these two strains, Wellcome and NF54/3D7 PfCSP, is high (94.21% identity, NCBI BLAST). In this study, 60% of mice vaccinated with the UPenn *PfCSP* mRNA-LNP1 were sterile protected, while 27% of the TriLink *PfCSP* mRNA-LNP1 at 3 doses versus 20% at 2 doses were protected (Fig. 4a). These findings reveal that, at least in the context of these experiments, two versus three doses at a 3-week interval were not significantly different for the *PfCSP* mRNA-LNP1 (TriLink), and similar to what was observed in BALB/c mice. Interestingly, an assessment of antibodies for in vitro inhibition in the ILSDA suggested a similar trend as the protective responses (Fig. 4b), with the greater inhibition induced by the UPenn mRNA at three doses and lesser with TriLink mRNA, at two doses. Additional experiments are ongoing to evaluate the relationship of these protective responses in mice and functional antibodies. While antibody responses to r-PfCSP paralleled functional antibodies (Fig. 4c–e), the relative differences in IgG2a:IgG1 ratios were significantly biased toward balanced responses with the UPenn *PfCSP* mRNA-LNP1 than with the three-dose TriLink *PfCSP* mRNA-LNP1 (Fig. 4f). Differences in cytokine levels induced in C57BL6 did not achieve significance with the exception of TNF-α (Th1 skew), IL-5 (Th2 skew), and the proinflammatory cytokines, KC/GRO and IL-10 (Supplementary Fig. 7).

## Discussion

As seen with the SARS-CoV-2 Pfizer/BioNTech and Moderna mRNA vaccines^39^, mRNA vaccines have numerous advantages over conventional vaccine approaches. Nominally, offering the advantage of exacting in silico antigen design, engagement of the host immune system through MHC Class I and II pathways, and the flexibility and rapidity of manufacturing processes. Intracellularly produced nucleic acid-delivered immunogens are capable of transferring antigen and/or nucleic acid to cells, including antigen-presenting cells for activation of the MHC class II pathway, resulting in antibody production^40,41^. In evaluating *PfCSP* mRNA as a malaria vaccine, the candidate was found to be well expressed in mammalian cells, immunogenic in mice, and protective in homologous and heterologous transgenic rodent models.

The literature reports that naked mRNA is suitable for in vivo expression when delivered by the intradermal (ID) route and by electroporation^42^. Delivery of 5 and 25 µg naked *PfCSP* mRNA (TriLink) by the ID route in a two-immunization regimen, yielded humoral and cellular responses that were below or near background levels of detection (data not reported). The failure to induce an immune response may be multifactorial and partially explained by unmodified mRNAs’ intrinsic susceptibility to extracellular ribonucleases, the challenges of negatively charged mRNAs to cross cellular membranes thus diminishing uptake, or mRNA sensing by the innate immune system. Others have reported similar findings, that unmodified mRNAs induce a robust type I interferon response, which reduces protein translation levels and enhances mRNA decay^43–45^. In any case, no further parameters were explored to address the lack of immunogenicity observed. Instead, to achieve the desired goal, LNP was applied to protect the *PfCSP* mRNA from extracellular ribonucleases, to facilitate efficient cell uptake through endocytosis, and to supply adjuvant activity that stimulates T follicular helper cells ^25^.

Currently, multiple formulations of four-component lipid nanoparticles are approved for use^46–48^ or in clinical trial evaluations^49^. Here, we evaluated several LNP formulations encapsulating nucleoside unmodified *PfCSP* mRNA (TriLink) for their ability to mount immune responses in BALB/c mice. Based on humoral and cytokine responses, only LNP1 advanced to more comprehensive analyses including protection studies in rodent malaria models. Short-term sterile protection was achieved with both high and low doses of *PfCSP* mRNA-LNP1 (TriLink), at 40% and 20% efficacy, respectively, against a lethal rodent infection with *P. berghei P. falciparum* CSP NF54/3D7 transgenic sporozoites. While not directly correlated, the high dose groups tended to have higher titers against the r-PfCSP and the repeat domain (NANP), along with the increased efficacy. While both anti-CSP antibodies and cell-mediated responses have been associated with protection using various vaccine platforms, no established correlate of protection exists for malaria. Rather than antibody titer, qualitative functional humoral responses more robustly predict protection from infection across vaccine regimens. These antibodies are likely essential and inhibit sporozoite entry into target hepatocytes, while cell-mediated immunity functions to support the humoral response, and possibly acts as an effector function against infected hepatocytes, although taken altogether; no direct evidence exists for the latter. To this end, a deeper dissection of the correlative value of the in vitro functional antibody inhibition observed in the ILSDA is being explored.

In the evaluation of a prototypic vaccine, such as the *PfCSP* mRNA-LNP (TriLink), an optimal immunization schedule was deduced empirically, and further guided by practical considerations. Refinements to the regimen were based on achieving relevant functional immunity. The goal of the present study was to establish optimal immune responses in mice based on dose and schedule using the mRNA-LNP platform. Results from other infectious disease research areas found that a single dose of mRNA-LNP is sufficient for inducing potent and functional immunity for certain pathogens^24,26,50^. From a regulatory and implementation standpoint, a single-dose vaccine is highly appealing. Considering this, we found that a single, high dose “prime” immunization of *PfCSP* mRNA-LNP (TriLink) induced responses that, while measurable, were inadequate for eliciting the high levels of functional antibody and cellular responses detected in the twice-dosed groups. These findings are not unique to malaria, since others have reported similar lower immunogenicity with a single dose mRNA-LNP, i.e., encoding ZIKV prM-E genes^32^. The disparity between these underwhelming results and data with successful outcomes using a single-dose strategy may be explained by the intrinsic nature of the target antigen, the mRNA transcript sequence, the mRNA dose, or other features not explored in this report.

Since mRNA mediates a rapid, albeit transient expression of the encoded protein, the magnitude and durability of antigen expression are critical to the induced immune response. Pardi et al. showed, in a kinetic study, using intramuscular injection of 1-methylpseudourindine 5′-nucleoside coding firefly luciferase mRNA encapsulated in LNP, that protein was detected in the tissue for several days to over a week with expression duration positively correlating with dose^22^. Under the conditions of the current study, a nucleoside-unmodified, codon harmonized full-length *PfCSP* mRNA-LNP1 (TriLink) and nucleoside-modified, codon-optimized *PfCSP* mRNA-LNP1 (UPenn) expressed PfCSP protein in transfected cells for at least 48 h in vitro. Depending on the duration of protein expression in vivo, increasing the dosing interval and the availability of antigen could lead to favorable antibody responses, through antibody production and B cell stimulation in germinal centers. Conventionally, while a 3-week or a 4-week dosing interval is not uncommon in preclinical studies^24,25,32,51^; a direct comparison of the two conditions has not been previously reported. Not surprisingly, a statistical evaluation of responses induced by the 3- versus 4-week intervals, two-dose groups, revealed no significant differences in immunogenicity, suggesting that this difference in interval was not sufficient to allow for possible contraction of activated cells. However, a trend was observed toward higher titer averages and higher inhibition in the ILSDA for the two doses, 4-week interval. These findings warranted an evaluation of longer intervals between the prime and boost as a mediator of improved efficiency of the boosting response. To this end, a three doses, 3-week versus 6-week interval in BALB/c mice compared the nucleoside-modified *PfCSP* mRNA (UPenn) and an unmodified mRNA of the same coding sequence (TriLink). We observed an improvement in protective responses that translated to 88% sterile protection (UPenn) and an antibody response that was biased to NANP repeat versus the C-terminal α-TSR. These findings support other studies where the magnitude of responses to NANP-repeat are at least partially associated with protection against infection ^35,52^.

An analysis of IgG subclasses revealed that the two-dose schedule yielded a relatively balanced IgG1 and IgG2a subclass profile with no significant differences detected by mRNA dose. Of interest, unlike the humoral responses, the cellular responses measured by in vitro stimulation of splenocytes were highly Th1 biased with marginal levels of IL-4 and IL-5, but potent IFN-γ, TNF-α, IL-2, and IL-12p70 responses. This may be explained by the high level of cytokine pathway complexity where cytokines serve as both activators, as well as immune modulators. For example, IFN-γ and its downstream effects may serve to modulate IL-4 production and vice versa^53–56^. These effects may be amplified under the cytokine assay conditions, since cytokines are allowed to accumulate over time rather than in the natural tissue environment where cytokine clearance and diffusion occur more readily. Notably, similar findings have been reported for other mRNA vaccines, that is, balanced IgG1/IgG2a ratios and/or high levels of Th1 biased cytokine production^50,51,57–60^. Administering a third 10 µg dose of *PfCSP* mRNA-LNP1 significantly increased the IgG2a subclass response compared to the two-dose regimen, while retaining the relative balance in the IgG2a to IgG1 ratio. This may be explained by mature B lymphocytes engagement in class-switch recombination (CSR) to produce a specific isotype and/or subclass antibody. This recombination event is highly regulated by the network of cytokines, which may have a Th1 or Th2 biased function and provide an indication of the immune skew. In the case of IgG1 and IgG2a, IL-4 enhances CSR to IgG1 production and IFN-γ to IgG2a^61,62^. The increase of IgG2a relative to IgG1 with a third dose of *PfCSP* mRNA-LNP1 suggests that the additional dose promoted Th1 skewed responses and drove CSR towards IgG2a production. Reports from a variety of pathogens have found that an IgG subclass balance or bias can have a marked effect on disease progression and immunity^63–67^. When specifically examining parasitic infections, positive outcomes correlate with balanced or Th1-skewed subclasses, much like the responses elicited by *PfCSP* mRNA-LNP1^63,67–69^. In addition to a subclass profile that shifted to a Th1 skew, a third dose of *PfCSP* mRNA-LNP1 resulted in significantly improved antibody titers to r-PfCSP and the NANP repeat peptide.

Despite numerous challenges, significant progress has been made toward the development of protective vaccines against pre-erythrocytic malaria infection. Many vaccine approaches have been evaluated in preclinical animal models, with few advancing into the clinic or beyond Phase 1 studies^70^. Encouraged by the successes of mRNA vaccines against other infectious diseases^17^, we applied an analogous approach to the age-old problem of malaria. We evaluated the effect of external factors such as dose, schedule, nucleoside modified versus unmodified mRNA and number of immunizations on PfCSP induced immunity and demonstrated the protective potential of a *PfCSP* mRNA-LNP against lethal, rodent-malaria transgenic parasites. Endogenous modifications on mRNA including nucleoside modifications, UTR length, codon content, microRNA target sites, and coding sequences, to name a few, can lead to improved mRNA stability and half-life, extending total protein production, and increasing interactions between antigen and the immune system^71–75^. As such, mRNA vaccines offer a versatile platform for optimization and refinement of sequences through an iterative process of engineering and reengineering. These results, applying the mRNA-LNP vaccine platform, are compelling, and lay the foundation for continued research efforts to enhance immunogenicity, protective efficacy, and the durability of responses to malaria.

## Methods

### mRNA design

The mRNA construct used in this study is based on the PfCSP sequence of the *P. falciparum* 3D7 strain and includes 1/4/38 copies of NPDP/NVDP/NANP motifs, respectively. The coding sequence (NCBI Reference Sequence: XM_001351086.1) was codon harmonized^76^ for optimal expression in mice, and included the native signal sequence but lacked the glycosylphosphatidylinositol (GPI) anchor sequence. *PfCSP* mRNA (TriLink) was synthesized using non-modified nucleosides, used the CleanCap^®^ capping method, included proprietary 5′ UTR, 3′ UTR sequence information, and was purified by silica membrane by TriLink BioTechnologies. A second *PfCSP* mRNA (Cap1-TEV) (UPenn) was identical in coding sequence as the above, but was codon-optimized for mRNA expression and cloned into the mRNA production plasmid as described^77^. mRNA production and LNP encapsulation was performed as described^77^. Briefly, mRNAs were transcribed to contain 101 nucleotide-long poly(A) tails. m1Ψ-5′-triphosphate (TriLink) instead of UTP was used to generate modified nucleoside-containing mRNA. Capping of the in vitro transcribed mRNAs was performed co-transcriptionally using the trinucleotide cap1 analog, CleanCap^®^ (TriLink). mRNA was purified by cellulose purification, as described^78^. All mRNAs were analyzed by agarose gel electrophoresis and were stored frozen at either −20 °C (UPenn) or −80 °C (TriLink).

### Cell lines

Chinese hamster ovary (CHO) E77.4 cells^79^ from ATCC were cultured in RPMI-1640 medium (Quality Biological 112-025-101) containing 10% heat-inactivated fetal bovine serum (Gibco™ 10082147), 2mM L-Glutamine (Quality Biological 118-084-721), and 100 U/mL Penicillin with 100 µg/mL Streptomycin (Quality Biological 120-095-721). Passages were performed using 0.05% Trypsin-0.1% EDTA (Quality Biological 118-087-721).

### Ethics statement

All animal procedures were conducted per the Institutional Animal Care and Use Committee (IACUC) at Walter Reed Army Institute of Research, Silver Spring, MD. This material has been reviewed by the Walter Reed Army Institute of Research. There is no objection to its publication. Research was conducted in an AAALACi accredited facility in compliance with the Animal Welfare Act and other federal statutes and regulations relating to animals and experiments involving animals and adheres to principles stated in the Guide for the Care and Use of Laboratory Animals, NRC Publication, 2011 edition.

### Animal studies

All animal studies used 5–6 week old female BALB/cJ or C57BL6 mice (The Jackson Laboratory). Mice were immunized by intramuscular injection in the posterior thigh muscle. Blood samples were collected by lateral tail bleeds one day prior to each immunization or challenge or by cardiocentesis two weeks following the final immunization. To determine protection against infection, mice were challenged two weeks following the final immunization. This study reports challenges with a *Plasmodium berghei* ANKA transgenic strain expressing the NF54/3D7 strain version of PfCSP (Pb-PfCSP; RMgm 4110, www.pberghei.eu), allowing for a homologous challenge^80^. The characterization of the *P. berghei* transgenic for the *P. falciparum* CSP NF54/3D7 strain was reported by Triller et al.^80^. C57BL6 mice were challenged with the *P. berghei* PfCSP Wellcome strain^81,82^. Sporozoites were harvested from the salivary glands of infected *Anopheles stephensi* mosquitoes using the Ozaki method and suspended in RPMI-1640 supplemented with 10% mouse serum^83^. The challenge dose was 1000 sporozoites by intravenous inoculation. In this study, protection was defined as the lack of blood-stage parasite detection two weeks following challenge. Parasites were observed using microscopy with thin blood smears fixed with methanol and stained with 10% Giemsa for 15 min at room temperature (Sigma Aldrich GS500). Mice were monitored for 2 weeks following the challenge. Vaccine efficacy was calculated using the formula: Efficacy = [1 − [(number of infected animals (*I*)_vaccine_/total number of animals (*n*)_vaccine_) ÷ (number of infected animals (*I*)_control_/total number of animals (*n*)_control_)]]*100.

### mRNA transfection

Transfections were performed according to the TransIT-mRNA protocol (Mirus Bio MIR 2225). Cells were plated at 300,000 cells/mL in a 24-well tissue culture plate for western blot detection and 80,000 cells/mL in an eight-well Nunc™ Lab-Tek™ II Chamber Slide™ System (Thermo Fisher 154534) for immunocytochemistry. Cells were incubated at 37 °C with 5% carbon dioxide for 24 h. Following this incubation, the cells were transfected with 0.5 µg/well mRNA or 1.0 µg/well mRNA-LNP for western blot detection or 0.18 µg/well mRNA for immunocytochemistry, then returned to 37 °C with 5% carbon dioxide. Transfected cells were harvested at 8, 24, and 48 h for western blot detection or at 16 h for immunocytochemistry. A negative control with TransIT reagents alone (no mRNA) was included to provide background detection levels.

### Western blot detection of PfCSP from mRNA transfected cells

Transfection harvests for western blot analysis included cell counting and normalization of the sample in regards to cell load. Following harvest and normalization, the samples were assessed using the Tris-Glycine SDS-PAGE system (Novex™) with SeeBlue™ Pre-stained Protein Standard, then transferred onto a nitrocellulose membrane for protein detection by western blot. Following transfer, the membrane was blocked with 5% non-fat dried milk (w/v) in PBS, pH 7.4 with 0.1% Tween-20 (PBS-T) (w/v) for 1 h at room temperature with mild agitation. The blot was washed three times with PBS-T for 5 min at room temperature with mild agitation between each step. The primary antibody (polyclonal rabbit serum generated against recombinant PfCSP) diluted in PBS-T at a ratio of 1:10,000 (antibody: diluent). The primary antibody incubation was 1 h at room temperature with gentle agitation. Following washes, the blot was probed with alkaline phosphatase-conjugated goat anti-rabbit IgG (Southern Biotech 4030-04) diluted in PBS-T at a ratio of 1:10,000 (antibody: diluent). Colorimetric detection was performed using 5-bromo-4-chloro-3-indolyl-phosphate with nitro blue tetrazolium (Sigma Aldrich 11383221001, 11383213001, respectively). The reaction was stopped with deionized water.

Densitometry analysis of western blot images was performed using ImageJ software^84^. Relative quantification of the bands was performed using the 10 ng recombinant PfCSP (r-PfCSP) band for each blot to account for blot-to-blot variation. The area selected for the 10 ng r-PfCSP was the major band at about 50 kDa. The r-PfCSP migrates lower than the transfected protein product because it lacks the native N-terminal 1-26 amino acids and has a reduced number of repeats (19 NANP + 3 NVDP) relative to the native protein (38 NANP + 4 NVDP), as reported by Genito et al^8^. . The area selected for evaluation of the experimental lanes was the major band detected at ~60 kDa and the doublet detected above this major band, likely an alternate oxidized form of PfCSP. The densitometry values calculated in the analogous area of the mock conditions were used to calculate background values, which were subtracted from the experimental condition values.

### Immunocytochemistry of cells transfected with *PfCSP* mRNA

Cell fixation occurred 16 h following transfection with 4% paraformaldehyde in phosphate-buffered saline, pH 7.4 (PBS) for 15 min. Slides were frozen at −40 °C until analyzed by immunocytochemistry. Slide wells were blocked with PBS with 1% bovine serum albumin (BSA) weight by volume (w/v). Three brief washes were performed between each step using PBS. Wells were probed with mouse monoclonal antibody 2A10 anti-*Plasmodium falciparum* CS repeat obtained through BEI Resources (NIAID, NIH: Monoclonal Antibody 2A10 Anti-*Plasmodium falciparum* Circumsporozoite Protein produced in vitro, MRA-183A, contributed by Elizabeth Nardin) diluted in PBS with 1% BSA to 10 µg/mL. Secondary detection was accomplished with 5 µg/mL fluorescein isothiocyanate (FITC) conjugated Goat anti-Mouse IgG (Southern Biotech 1034-02) supplemented with 100 µg/mL of 4′,6-diamidino-2-phenylindole (DAPI) in PBS with 1% BSA. Following final washes, slides were allowed to air dry under reduced light, then mounted using Fluoromount-G (Southern Biotech 0100-01). Images were acquired using a BX53F microscope and DP80 camera (Olympus). Microscope settings (exposure time, bulb intensity) were consistent across all images within an experiment. Images were acquired at 63× magnification. For fluorescence area detection, the FITC and DAPI fluorescent areas (µm^2^) were measured across twenty fields at ×25.2 magnification for each condition using Olympus cellSens imaging software. To account for the varied size and quantity of cells between fields, FITC measurements were normalized to the DAPI measurements within the same field, resulting in a FITC/DAPI area ratio. Background levels of this ratio were detected under mock conditions where the cells were only exposed to the transfection reagents, not the mRNA. Commercially obtained antibodies were used according to manufacturer’s instructions.

### mRNA formulation in LNP

Lipid nanoparticles used in this study were similar in composition to those previously described^85,86^ and contain ionizable lipids proprietary to Acuitas (pKa in the range of 6.0-6.5)/DSPC/Cholesterol/PEG-Lipid). Encoding mRNA was encapsulated in LNP using a self-assembly process in which an aqueous solution of mRNA at 4.0 pH was rapidly mixed with a solution containing a premix of the aforementioned lipids premixed and dissolved in ethanol. The proprietary lipids and LNP composition are described in US patent applications WO 2017/075531 and WO 2017/0041443. All LNP were characterized post-production at Acuitas Therapeutics (Vancouver, BC, Canada) for their size and polydispersity (PDI) using a Malvern Zetasizer (Zetasizer Nano DS, Malvern, UK) and encapsulation efficiency using ribogreen (RG)^87^. Characterization results were measured and calculated using Malvern Panalytical Software (Malvern, UK) are listed here: LNP1—size range: 68–75 nm, PDI: < 0.054, RG: 95–97%; LNP2—size range: 64 nm, PDI: < 0.061, RG: 95%; LNP3—size range: 70 nm, PDI: < 0.095, RG: 86%. All mRNA-LNPs were stored at −80 °C.

### Enzyme linked immunosorbent assay (ELISA) for PfCSP detection (r-PfCSP, (NANP)_6_ peptide, and Pf16 (α-TSR peptide))

Briefly, 96-well 2HB plates (Immulon 3455) were coated with the recombinant nearly full-length PfCSP (r-PfCSP) at 100 ng/well or (NANP)_6_ peptide at 20 ng/well in PBS overnight at 4 °C^8,82,88^. For C-terminal peptide (α-TSR) detection, streptavidin plates (Greiner Bio One 655990) were coated at 100 ng/well. The r-PfCSP purification process was previously reported by Schwenk et al^89^. . The (NANP)_6_ peptide (H2N-NANPNANPNANPNANPNANPNANPC-COOH) and Pf16 peptide (Biotin-AHX­EPSDKHIKEYLNKIQNSLSTEWSPCSVTCGNGIQVRIKPGSANKPKDELDYANDIEKKICKMEKCS­NH2) were purchased from a commercial vendor (Biomatik). Wells were blocked with PBS with 1% BSA for 1 h at 22 °C. Initial dilutions of sera were generated in PBS with 1% BSA, followed by serial dilutions on the 96-well plate. The primary antibody incubation was for 2 h at 22 °C. The secondary antibody (HRP conjugated goat anti-mouse IgG [KPL 074-1806]) was diluted in PBS with 1% BSA at 1:4000 (antibody: diluent) and 100 µL added to all wells. The secondary antibody incubation was for 1 h at 22 °C. Development was performed using ABTS Peroxidase Substrate 2-Component System (KPL 5120-0032) for 1 h at 22 °C, then detected using an M2 spectrophotometer (Molecular Devices) at 415 nm wavelength. Between steps, all wells were washed three times with 250 µL wash buffer (PBS, 0.05% Tween 20, 0.1% chlorohexidine [w/v]) using a BioTek Microplate Washer 405. Positive and negative controls were included on each plate. Midpoint titers detected by ELISA were defined as the dilution required to achieve an OD_415_ = 1. Of note, the lower limit of detection for this assay is 1:400. If a sample does not achieve a calculable midpoint titer (OD_415_ = 1) at a 1:400 dilution, the sample data is reported as below the limits of detection.

### Avidity ELISA

Avidity ELISAs were performed as described above with a chaotrope step incorporated after the primary antibody step. For each sample plate, an identical plate was generated. Following the post-primary antibody washes, one duplicate plate was treated with PBS and the other was treated with a chaotrope solution (1.5 M sodium thiocyanate in PBS). The chaotrope incubation was at 22 °C for 30 min. The avidity index was calculated with this equation: ((OD 1 titer in chaotrope solution/OD 1 titer in PBS)*100). Samples that did not reach a midpoint titer (OD_415_ = 1) at a 1:400 dilution by the r-PfCSP ELISA method were not included in avidity analyses.

### IgG subclass ELISA

IgG subclass ELISAs were performed as described in the ELISA for r-PfCSP at 100 ng/well and adjusted secondary antibody conditions. Each mouse serum sample was assayed on three separate plates for IgG subclass detection of IgG1, IgG2a, or IgG2b. The secondary antibody (HRP conjugated goat anti-mouse IgG1, IgG2a, and IgG2b [Southern Biotech 1070-05, 1080-05, and 1090-05, respectively]) was diluted in PBS with 1% BSA at 1:4000 (antibody: diluent) and 100 µL added to all wells.

### Inhibition of liver stage development assay (ILSDA)

ILSDA methods were performed as previously described^90^. All serum samples were diluted at 1:40. Final data were reported as percent inhibition. This value was calculated by relating the level of *Plasmodium falciparum* 18S rRNA detection in the experimental sample to the calculated average of 18S rRNA detection in a condition, where sporozoites were incubated with human hepatocytes in the absence of mouse serum. Navy falciparum sporozoite 1 (NFS1), an IgG1 monoclonal antibody to the *P. falciparum* CSP major repeat served as the positive control for ILSDAs and was generated by the Naval Medical Research Center for this purpose.

### Enzyme linked immunospot (ELISpot) assay

Hydrophobic 96-well plates with 0.45 µm pore size PVDF membranes (EMD Millipore S2EM004M99) were coated with capture antibody according to the manufacturer’s instructions for the Mouse IFN-γ ELISpot assay (R&D Systems SEL485). The stimulating antigen was 1 µg/mL PfCSP (3D7) overlapping 15-mer peptide pool. The positive control for cell stimulation was 1 µg/mL hamster anti-mouse CD3e (BD Biosciences 553057). The negative control was culture media alone. Plates were blocked with complete media for at least 2 h. Mouse splenocytes were plated based on spot counting optimization and ranged from 25,000 to 100,000 cells/well. Plates were incubated for 42 h at 37 °C with 5% carbon dioxide for cell stimulation. Wells were probed with the detection antibody according to the manufacturer’s instructions. Following the detection incubation, plates were developed with the ELISpot Blue Color Module according to the manufacturer’s instructions (R&D Systems SEL002) and allowed to dry completely before analysis. Spot counting was performed using an AID ELISpot Reader (Autoimmune Diagnostika).

### Cytokine detection by Meso Scale Discovery

Mouse splenocytes were plated at 400,000 cells/well in 96-well flat bottom plates (Costar 3595). The stimulating antigen was 1 µg/mL PfCSP (3D7) overlapping 15-mer peptide pool. Cells were stimulated for 48 h by incubation at 37 °C with 5% carbon dioxide. Cell culture supernatant was harvested and pro-inflammatory cytokines measured using the V-PLEX Plus Mouse IFN-y Kit (Meso Scale Discovery K152QOG-2) and V-PLEX Proinflammatory Panel 1 Mouse Kit (Meso Scale Discovery K15048D) according to the manufacturer’s instructions. Cytokine levels detected with cells incubated in culture media alone served to normalize for background cytokine secretion.

### Reporting summary

Further information on research design is available in the Nature Research Reporting Summary linked to this article.

## Supplementary information

Supplementary Information

Reporting Summary

## Data Availability

The datasets generated and/or analyzed during the current study are available from the corresponding author on reasonable request.
