# Supplementary material for: Messenger RNA expressing PfCSP induces functional, protective immune responses against malaria in mice
Source: NPJ Vaccines. 2021 Jun 18;6:84. doi: 10.1038/s41541-021-00345-0 (PMC8213722; doi:10.1038/s41541-021-00345-0)
Supplement: Supplementary file 1 — Supplementary Information [file 41541_2021_345_MOESM1_ESM.pdf]

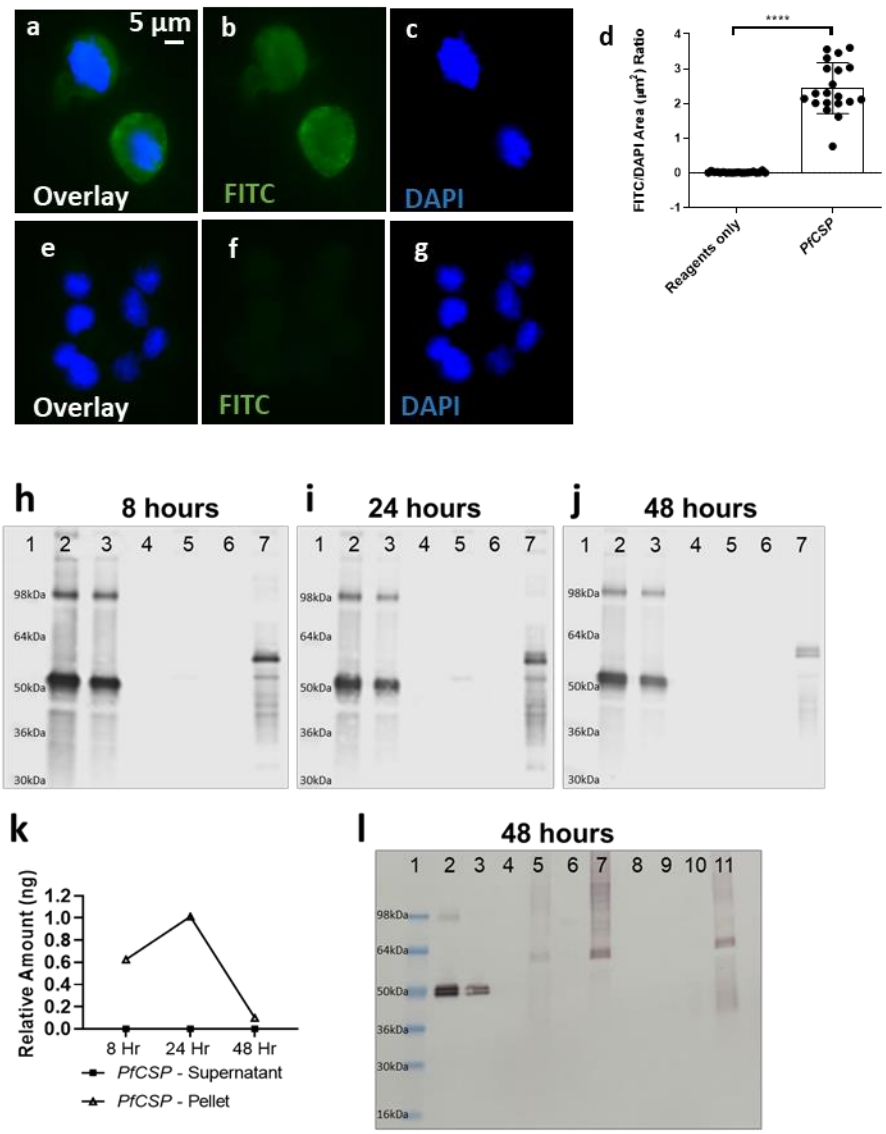

Supplementary Figure 1. PfCSP protein expression in CHO cells following *in vitro* transfection with *PfCSP* mRNA. a) Overlay of PfCSP protein expressed by CHO cells with the nuclear stain. The protein was immunolabeled with FITC using 2A10 mouse monoclonal antibody, shown in green and DAPI to view the nucleus, shown in blue. The raw FITC b) and DAPI c) images are depicted. d) Images were analyzed for the area of fluorescence detection for DAPI and FITC. To effectively normalize the amount of PfCSP detection to the number and size of cells in the field, the area of PfCSP detection (FITC) within a field was calculated relative to the area of nuclear stain (DAPI) within a field and reported as a ratio. d). The difference in PfCSP detection using the 2A10 monoclonal between the cells transfected with *PfCSP* mRNA (TriLink) and the cells exposed to transfection reagents alone was found to be highly significant in an unpaired t test (**** = $p<0.0001$), mean and standard deviation. Panel e-g) Negative control panels, under identical transfection and detection conditions, in the absence of *PfCSP* mRNA. Panel h-j) Western blot analysis using rabbit polyclonal serum were performed on transfected CHO cell culture supernatants and pellets to assess localization of PfCSP. Harvests were performed at h) 8, i) 24, and j) 48

hours following transfection. Lane 1 in all panels is the SeeBlue molecular weight marker. Lanes 4 and 5 represents the supernatant and pellet fraction for negative control (no mRNA), respectively. Lanes 6 and 7 represents the supernatant and pellet fraction for CHO cells transfected with *PfCSP* mRNA (TriLink), respectively. The 30ng and10ng of r-PfCSP served as a positive blotting and detection control (Lanes 2 and 3, respectively). k) Relative density of the major band in the transfection conditions was calculated using the 10ng r-PfCSP reference band, as an approximation of the amount of protein detected in the pellet fractions. All blots in Panels h-j, were derived from the same experiment and were processed in parallel. Western blot analysis was also performed to assess the transfection efficiency and expression of *PfCSP* mRNA-LNPs. CHO cells were transfected with *PfCSP* mRNA-LNP1 (TriLink) and *PfCSP* mRNA-LNP1 (UPenn). l) Sample harvests were performed at 48 hours post transfection. Lanes 2 and 3 represents the 10ng and 3ng r-PfCSP as detection controls, respectively. Lanes 4 and 5 represents the supernatant and pellet fraction of CHO cells transfected with *PfCSP* mRNA-LNP1 (TriLink) in TransIT reagent alone. Lanes 6 and 7 represent the supernatant and pellet fractions of CHO cells transfected with *PfCSP* mRNA-LNP1 (UPenn) in TransIT reagent alone. Lanes 8-11 serves as a controls. Lanes 8 and 9 represents the supernatant and pellet fractions of CHO cells transfected with *PfCSP* mRNA (TriLink) (in Boost reagent only), while lanes 10 and 11 represent the supernatant and pellet fractions of CHO cells transfected with *PfCSP* mRNA (TriLink) (in TransIT and boost reagents), respectively. Panel l was derived from a separate experiment and processed separately from Panel h-j.

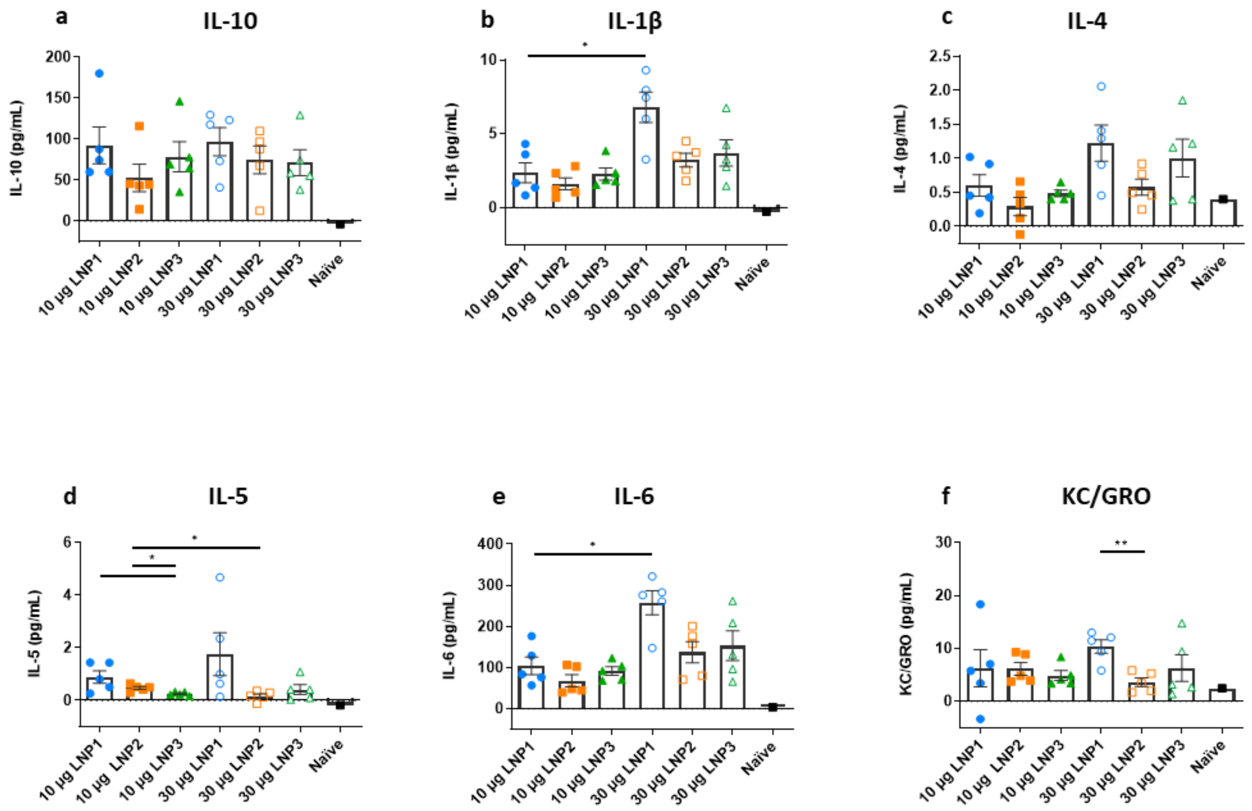

Supplementary Figure 2. Secreted cytokine concentrations in BALB/c immunized with *PfCSP* mRNA (TriLink) encapsulated in three LNPs (LNP1, LNP2 and LNP3), at two mRNA doses (10µg and 30µg). Splenocytes were incubated in the presence of a PfCSP overlapping 15-mer peptide pool. Cell culture supernatants were harvested after 48 hours and cytokine concentrations quantified by MSD. (N=5 per group) a) IL-10 b) IL-1β, c) IL-4, d) IL-5, e) IL-6 and f) KC/GRO. Data are reported as the mean and standard error of the mean (SEM), in pg/mL. All statistical analyses in this figure were performed using a Mann Whitney test (* = p<0.05; ** = p<0.01).

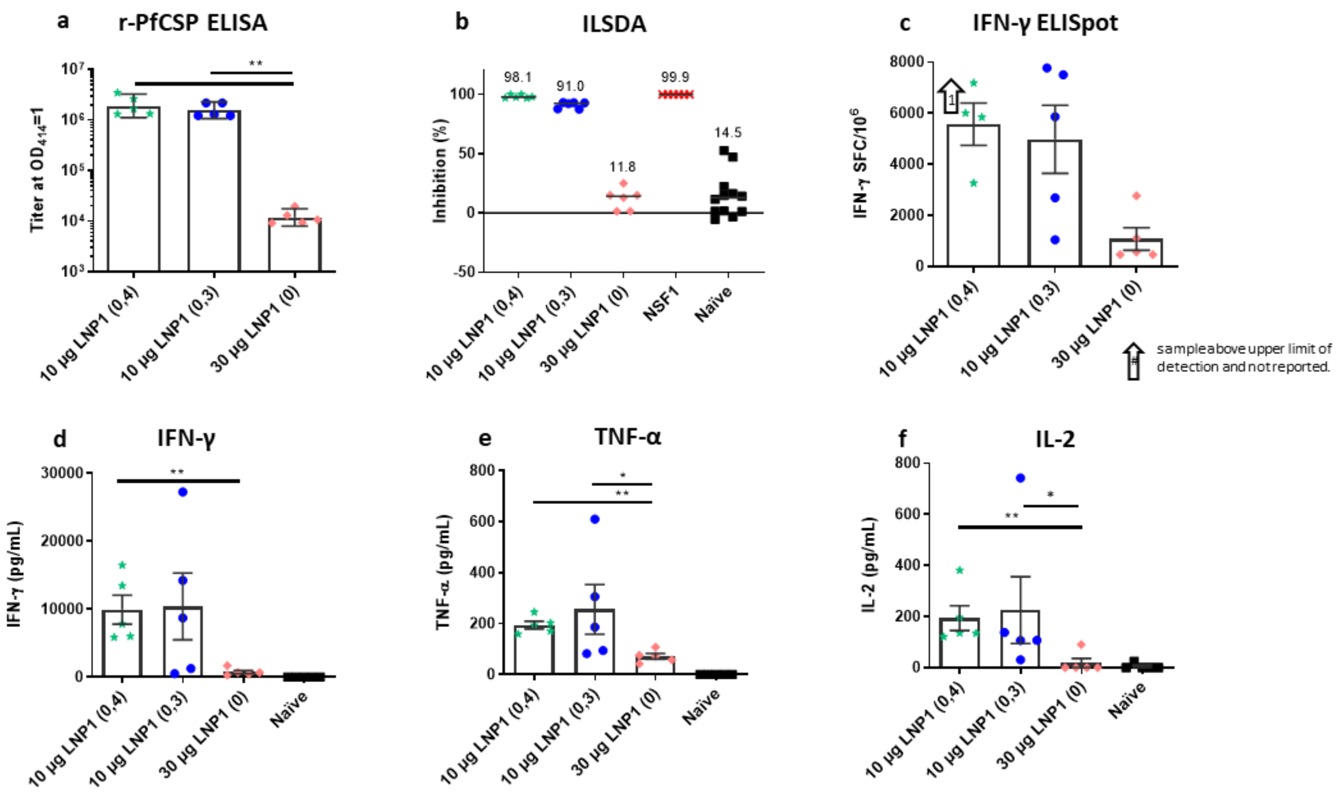

Supplementary Figure 3. A single, high-dose prime of *PfCSP* mRNA-LNP induced inferior responses. BALB/c were immunized with either a four week interval low-dose prime:boost, a three week interval low-dose prime:boost, or a single high-dose prime regime (N=5 per group). a) PfCSP-specific antibody titers were assessed by r-PfCSP titration ELISA. Data are reported as the GM with 95% CI. b) Functional inhibition was evaluated by ILSDA. Data are reported as the mean of assay replicates. c) An IFN-γ ELISpot was used to detect splenocytes reactive to PfCSP peptides. Splenocytes were incubated in the presence of a PfCSP overlapping 15-mer peptide pool. Cells positive for IFN-γ production per million splenocytes were calculated by averaging the number of spots per sample replicate, then multiplying with the dilution factor to report the number of spot forming cells (SFC) per million splenocytes. Data are reported as the mean and SEM. For d) IFN-γ, e) TNF-α, f) IL-2, the cell culture supernatant of splenocytes incubated in the presence of PfCSP overlapping 15-mer peptide pool was evaluated for cytokine production using MSD (N=5). All statistical analyses were performed using a Mann Whitney test (*$p<0.05$, **$p<0.01$).

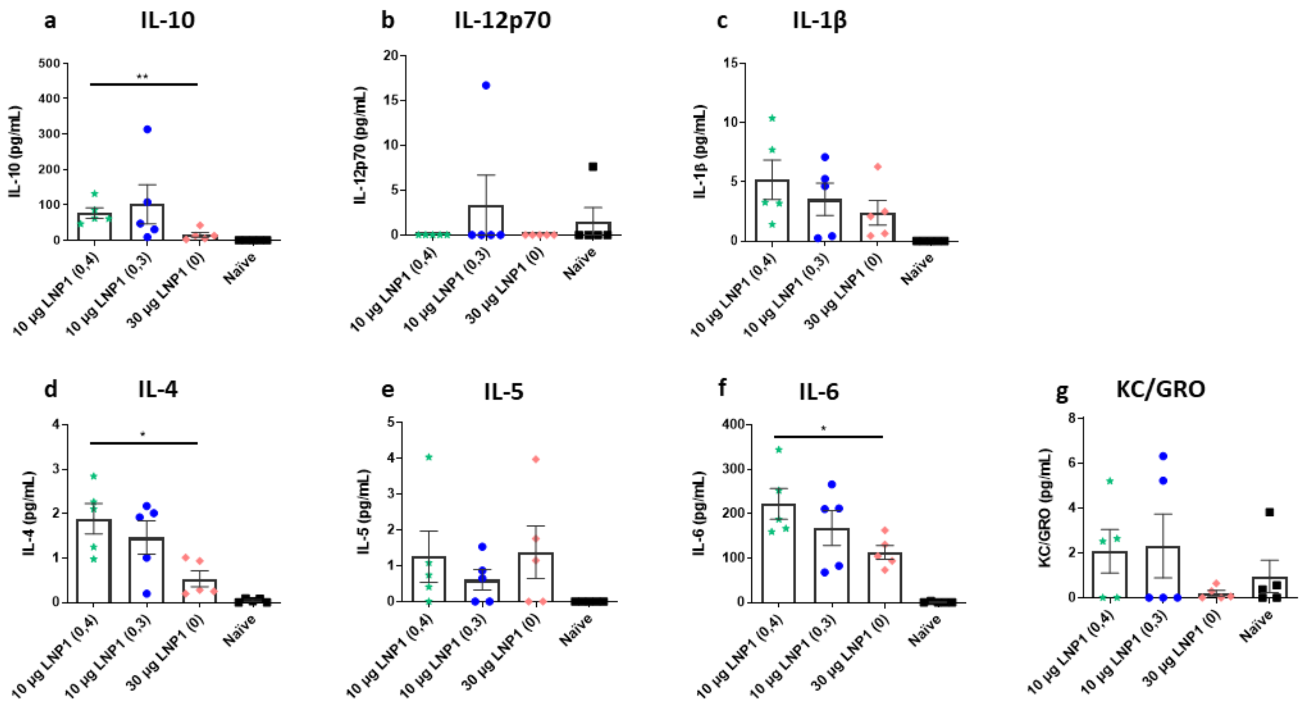

Supplementary Figure 4. Secreted cytokine concentrations in BALB/c immunized with *PfCSP* mRNA-LNP1 (TriLink) with mRNA doses (10µg and 30µg) and schedules (0,4; 0,3; and 0 week intervals). The cell culture supernatant of splenocytes incubated in the presence of PfCSP overlapping 15-mer peptide pool was evaluated for cytokine production using MSD. a) IL-10 b) IL-12p70, c) IL-1β, d) IL-4, e) IL-5, f) IL-6 and g) KC/GRO. Data are reported as the mean and SEM, in pg/mL (N=5 per group). All statistical analyses were performed using a Mann Whitney test (*p<0.05, **p<0.01).

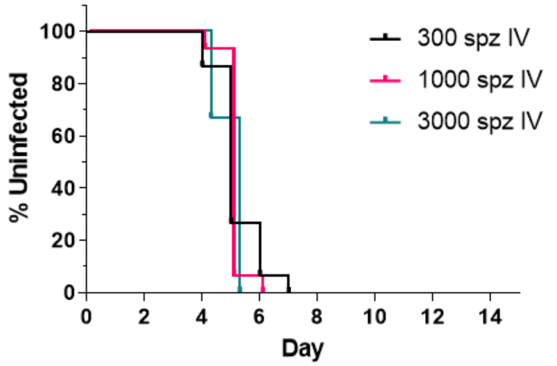

Supplementary Figure 5. BALB/c were injected in the lateral tail vein with 300, 1000, or 3000 *P. berghei* PfCSP NF54/3D7 transgenic sporozoites and monitored for blood parasitemia for 14 days following infection. Data represents the average of three independent experiments (N=5, per challenge dosage group, per experiment).

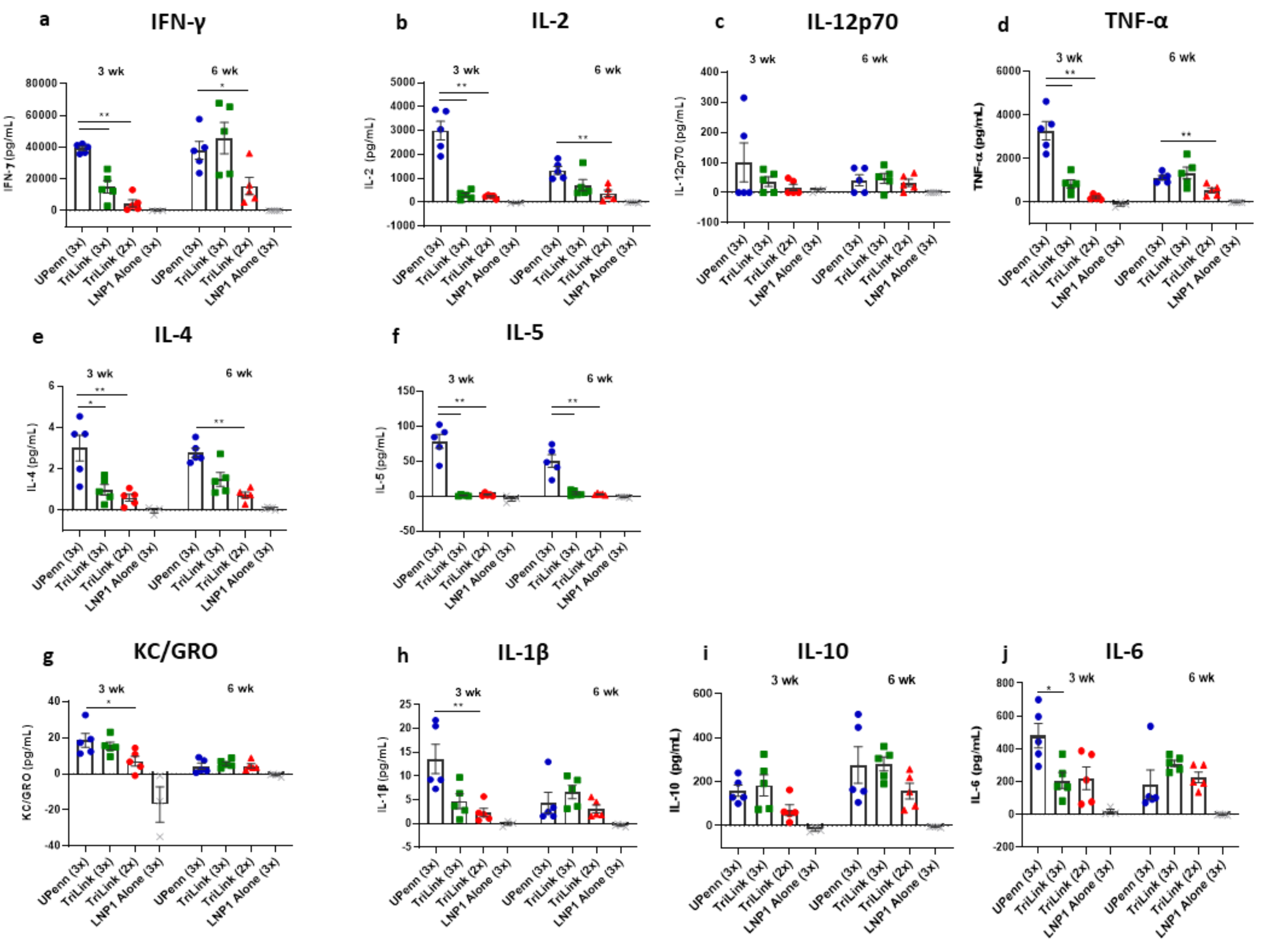

Supplementary Figure 6. Secreted cytokine concentrations in BALB/c immunized with *PfCSP* mRNAs-LNP1 (TriLink versus UPenn) in a 3 or 6 week interval schedule. The cell culture supernatant of splenocytes incubated in the presence of PfCSP overlapping 15-mer peptide pool was evaluated for cytokine production using MSD. Th1 cytokines a) IFN-γ b) IL-2, c) IL-12p70, d) TNF-α, Th2 cytokines e) IL-4, f) IL-5, and pro-inflammatory cytokines g) KC/GRO, h) IL-1β, i) IL-10, j) IL-6 . Data are reported as the mean and SEM, in pg/mL. N=5 per group, except LNP1 alone, N=3. All statistical analyses were performed using a Mann Whitney test (*p<0.05, **p<0.01).

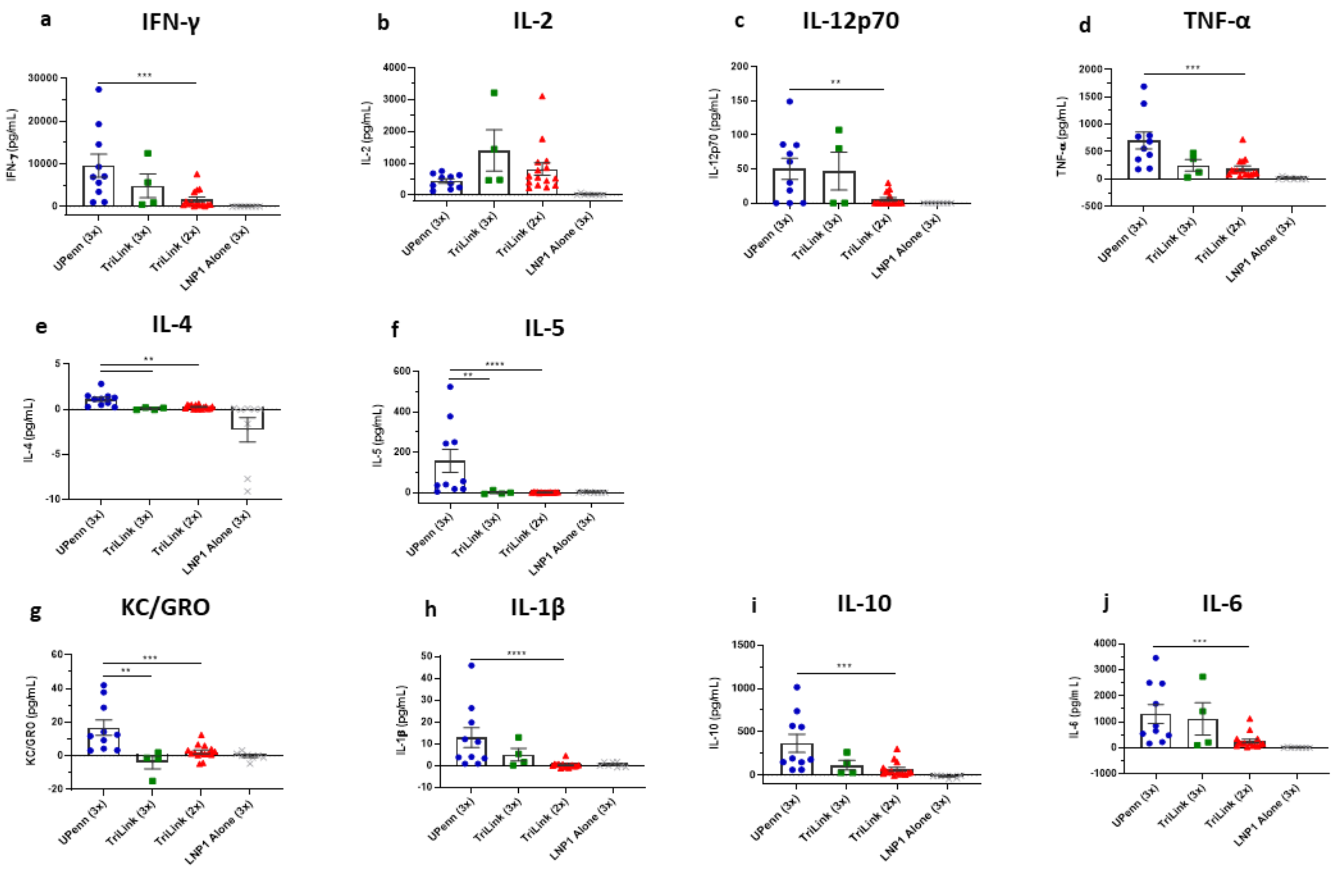

Supplementary Figure 7. Secreted cytokine concentrations in C57BL6 immunized with *PfCSP* mRNAs-LNP1 (TriLink versus UPenn). The cell culture supernatant of splenocytes incubated in the presence of PfCSP overlapping 15-mer peptide pool was evaluated for cytokine production using MSD. Th1 cytokines a) IFN-γ b) IL-2, c) IL-12p70, d) TNF-α, and Th2 cytokines e) IL-4, f) IL-5, and pro-inflammatory cytokines g) KC/GRO, h) IL-1β, i) IL-10, j) IL-6 . Data are reported as the mean and SEM, in pg/mL. Number of samples per group (N=10 UPenn 3x; N=4 TriLink 3x; N=15 TriLink 2x; N=8 LNP1 alone). All statistical analyses were performed using a Mann Whitney test (*p<0.05, **p<0.01, ***p<0.001, ****p<.0001).
