# Supplementary material for: Messenger RNA expressing PfCSP induces functional, protective immune responses against malaria in mice
Source: NPJ Vaccines. 2021 Jun 18;6:84. doi: 10.1038/s41541-021-00345-0 (PMC8213722; doi:10.1038/s41541-021-00345-0)
Supplement: Supplementary file 2 — Reporting Summary [file 41541_2021_345_MOESM2_ESM.pdf]

nature research

Corresponding author(s): Evelina Angov, Ph.D.

Last updated by author(s): May 19, 2021

Reporting Summary

Nature Research wishes to improve the reproducibility of the work that we publish. This form provides structure for consistency and transparency in reporting. For further information on Nature Research policies, see our Editorial Policies and the Editorial Policy Checklist.

Statistics

For all statistical analyses, confirm that the following items are present in the figure legend, table legend, main text, or Methods section.

n/a	Confirmed	
☐	☒	The exact sample size (n) for each experimental group/condition, given as a discrete number and unit of measurement
☐	☒	A statement on whether measurements were taken from distinct samples or whether the same sample was measured repeatedly
☐	☒	The statistical test(s) used AND whether they are one- or two-sided<br>*Only common tests should be described solely by name; describe more complex techniques in the Methods section.*
☒	☐	A description of all covariates tested
☐	☒	A description of any assumptions or corrections, such as tests of normality and adjustment for multiple comparisons
☐	☒	A full description of the statistical parameters including central tendency (e.g. means) or other basic estimates (e.g. regression coefficient) AND variation (e.g. standard deviation) or associated estimates of uncertainty (e.g. confidence intervals)
☐	☒	For null hypothesis testing, the test statistic (e.g. F, t, r) with confidence intervals, effect sizes, degrees of freedom and P value noted<br>*Give P values as exact values whenever suitable.*
☒	☐	For Bayesian analysis, information on the choice of priors and Markov chain Monte Carlo settings
☒	☐	For hierarchical and complex designs, identification of the appropriate level for tests and full reporting of outcomes
☐	☒	Estimates of effect sizes (e.g. Cohen's d, Pearson's r), indicating how they were calculated

Our web collection on statistics for biologists contains articles on many of the points above.

Software and code

Policy information about availability of computer code

Data collection	No code or software for data collection was used.
Data analysis	GraphPad Prism V.8.0<br>ImageJ Software

For manuscripts utilizing custom algorithms or software that are central to the research but not yet described in published literature, software must be made available to editors and reviewers. We strongly encourage code deposition in a community repository (e.g. GitHub). See the Nature Research guidelines for submitting code & software for further information.

Data

Policy information about availability of data

All manuscripts must include a data availability statement. This statement should provide the following information, where applicable:
- Accession codes, unique identifiers, or web links for publicly available datasets
- A list of figures that have associated raw data
- A description of any restrictions on data availability

The datasets generated and/or analyzed during the current study are available from the corresponding author on reasonable request.

Field-specific reporting

Please select the one below that is the best fit for your research. If you are not sure, read the appropriate sections before making your selection.

☒ Life sciences ☐ Behavioural & social sciences ☐ Ecological, evolutionary & environmental sciences

For a reference copy of the document with all sections, see nature.com/documents/nr-reporting-summary-flat.pdf

Life sciences study design

All studies must disclose on these points even when the disclosure is negative.

Sample size	In order to assure statistical significance for results from in vitro cellular assays, an N =5 mice per group was selected (samples from each mouse will be run in triplicate in the experiments, the experiments will be repeated at least once – depending on the amount of material available). This number can be confirmed when using power analysis (nQuery Advisor 6.0 software). In order to achieve a 80% (i.e. a power of 80%) chance to detect a statistical difference of size (2.0) between two means with a common standard deviation of (1.0) using a 2-group t-test (2-sided with alpha = 0.05) with equal variance, or using ANOVA with equal variances applying contrast option to compare the largest difference among the multiple groups, five mice per group will be required. In challenge experiments, the number of mice per group will increase to 10. We can assume that the meaningful effect size difference is about 1.5 and the variance is about 1, then using a contrast statement, if the number per group is 10, then the power achieves over 0.80.
Data exclusions	One instance of data exclusion is reported in the Results section. Due to the data being outside the range of the assay detection no statistical analysis was reported on this data set.
Replication	Experimental replicates are noted if present in the manuscript. N's for each dataset are reported in Figure legends. Each data set was analyzed against internal controls recognizing that some variance may exist due to differences in lots of mRNA, LNP, or mice litters between experiments.
Randomization	All animals were randomly assigned to vaccination groups, and were matched litter mates per experiment.
Blinding	Blinding was not performed, however, all experiments were carried out in an unbiased manner to prevent potential biases in the experimental groups.

Reporting for specific materials, systems and methods

We require information from authors about some types of materials, experimental systems and methods used in many studies. Here, indicate whether each material, system or method listed is relevant to your study. If you are not sure if a list item applies to your research, read the appropriate section before selecting a response.

Materials & experimental systems

n/a	Involved in the study	
☐	☒	Antibodies
☐	☒	Eukaryotic cell lines
☒	☐	Palaeontology and archaeology
☐	☒	Animals and other organisms
☒	☐	Human research participants
☒	☐	Clinical data
☒	☐	Dual use research of concern

Methods

n/a	Involved in the study	
☒	☐	ChIP-seq
☒	☐	Flow cytometry
☒	☐	MRI-based neuroimaging

Antibodies

Antibodies used	Polyclonal rabbit serum generated against recombinant PfCSP<br>Alkaline phosphatase conjugated goat anti-rabbit IgG (Southern Biotech 4030-04)<br>Mouse monoclonal antibody 2A10 obtained through BEI Resources (NIAID, NIH: Monoclonal Antibody 2A10 Anti-Plasmodium falciparum Circumsporozoite Protein produced in vitro, MRA-183A, contributed by Elizabeth Nardin)<br>Fluorescein isothiocyanate (FITC) conjugated Goat anti-Mouse IgG (Southern Biotech 1034-02)<br>HRP conjugated goat anti-mouse IgG [KPL 074-1806]<br>HRP conjugated goat anti-mouse IgG1, IgG2a, and IgG2b [Southern Biotech 1070-05, 1080-05, and 1090-05, respectively]<br>Navy falciparum sporozoite 1 (NFS1) generated by the Naval Medical Research Center<br>Mouse IFN-γ ELISpot assay capture and detection antibody (R&D Systems SEL485)<br>V-PLEX Plus Mouse IFN-γ detection antibody (Meso Scale Discovery K152QOG-2)<br>V-PLEX Proinflammatory Panel 1 Mouse detection antibodies (Meso Scale Discovery K15048D)
Validation	Commercial antibodies were used according to manufacturer's instructions and this is noted in the manuscript where appropriate.

Eukaryotic cell lines

Policy information about cell lines

Cell line source(s)	Chinese hamster ovary (CHO) E77.4 cells from ATCC
Authentication	Cell lines were not authenticated, were obtained from a commercial source with certificates of analysis.
Mycoplasma contamination	Cell lines were not tested for mycoplasma.
Commonly misidentified lines (See ICLAC register)	No commonly misidentified cell lines were used in this study.

Animals and other organisms

Policy information about studies involving animals; ARRIVE guidelines recommended for reporting animal research

Laboratory animals	5-6 week old, female BALB/c and C57BL/6 mice were used in these studies, obtained from The Jackson Laboratories.
Wild animals	Not applicable
Field-collected samples	Not applicable
Ethics oversight	All animal procedures were conducted per the Institutional Animal Care and Use Committee (IACUC) at Walter Reed Army Institute of Research, Silver Spring, MD. This material has been reviewed by the Walter Reed Army Institute of Research. There is no objection to its publication. Research was conducted in an AAALACi accredited facility in compliance with the Animal Welfare Act and other federal statutes and regulations relating to animals and experiments involving animals and adheres to principles stated in the Guide for the Care and Use of Laboratory Animals, NRC Publication, 2011 edition.

Note that full information on the approval of the study protocol must also be provided in the manuscript.
